# The Enantiomer of Allopregnanolone Prevents Pressure-Mediated Retinal Degeneration Via Autophagy

**DOI:** 10.3389/fphar.2022.855779

**Published:** 2022-03-16

**Authors:** Makoto Ishikawa, Toru Nakazawa, Hiroshi Kunikata, Kota Sato, Takeshi Yoshitomi, Kathiresan Krishnan, Douglas F. Covey, Charles F. Zorumski, Yukitoshi Izumi

**Affiliations:** ^1^ Department of Ophthalmic Imaging and Information Analytics, Tohoku University Graduate School of Medicine, Sendai, Japan; ^2^ Department of Ophthalmology, Tohoku University Graduate School of Medicine, Sendai, Japan; ^3^ Department of Retinal Disease Control, Tohoku University Graduate School of Medicine, Sendai, Japan; ^4^ Department of Advanced Ophthalmic Medicine, Tohoku University Graduate School of Medicine, Sendai, Japan.; ^5^ Department of Orthoptics, Fukuoka International University of Health and Welfare, Fukuoka, Japan; ^6^ Department of Ophthalmology, Akita University School of Medicine, Akita, Japan; ^7^ Department of Developmental Biology, Washington University School of Medicine, St. Louis, MO, United States; ^8^ Department of Anesthesiology, Washington University School of Medicine, St. Louis, MO, United States; ^9^ Taylor Family Institute for Innovative Psychiatric Research, Washington University School of Medicine, St. Louis, MO, United States; ^10^ Department of Psychiatry, Washington University School of Medicine, St. Louis, MO, United States; ^11^ Center for Brain Research in Mood Disorders, Washington University School of Medicine, St. Louis, MO, United States

**Keywords:** autophagy, allopregnanolone, enantiomer, glaucoma, intraocular pressure, neurosteroid

## Abstract

In an *ex vivo* rat ocular hypertension (OHT) model, the neurosteroid allopregnanolone (AlloP) exerts neuroprotective effects via enhancement of both GABA_A_ receptors and autophagy. We now examine whether its enantiomer (*ent-*AlloP), which is largely inactive at GABA receptors, offers similar neuroprotection in *ex vivo* and *in vivo* rat OHT models. *Ex vivo* rat retinal preparations were incubated in a hyperbaric condition (10 and 75 mmHg) for 24 h. An *in vivo* ocular hypertension (OHT) model was induced by intracameral injection of polystyrene microbeads. We examined pharmacological effects of AlloP, *ent-*AlloP, picrotoxin (a GABA_A_ receptor antagonist), and 3-MA (an autophagy inhibitor) histologically and biochemically. We found that both AlloP and *ent*-AlloP have marked neuroprotective effects in the retina, but effects of the unnatural enantiomer are independent of GABA_A_ receptors. Electron microscopic analyses show that pressure elevation significantly increased autophagosomes (APs) in the nerve fiber layer and addition of AlloP also increased APs and degenerative autophagic vacuoles (AVds). *ent*-AlloP markedly increased APs and AVds compared to AlloP. Examination of LC3B-II and SQSTM1 protein levels using immunoblotting revealed that AlloP increased LC3B-II, and *ent*-AlloP further enhanced LC3B-II and suppressed SQSTM1, indicating that autophagy is a major mechanism underlying neuroprotection by *ent*-AlloP. In an rat *in vivo* OHT model, single intravitreal *ent*-AlloP injection prevented apoptotic cell death of retinal ganglion cells similar to AlloP. However, even in this model, *ent*-AlloP was more effective in activating autophagy than AlloP. We conclude that *ent*-AlloP may be a prototype of potential therapeutic for treatment of glaucoma as an autophagy enhancer without affecting GABA receptors.

## Introduction

Glaucoma is one of the leading causes of irreversible blindness (Tham et al., 2014) and is characterized by selective degeneration of retinal ganglion cells (RGCs) (Quigley, 1999). Elevation of intraocular pressure (IOP) is considered as a significant risk factor for glaucoma (The AGIS Investigators, 2000). However, the molecular mechanisms of IOP induced RGC damage still remain elusive.

Neurosteroids are endogeneous steroids synthesized within the nervous system and can rapidly modulate neuronal excitability. The neurosteroid, allopregnanolone (AlloP) is a potent and effective positive allosteric modulator of GABA_A_ receptors (Locci and Pinna 2017). We previously reported that AlloP protected RGC from pressure-induced retinal injury in *ex vivo* rat retinas (Ishikawa et al., 2014, 2016). Because the neuroprotection by AlloP was inhibited by a specific GABA_A_ antagonist, the neuroprotective effect of AlloP seemed likely mediated by GABAergic signaling.

However, neuroprotection by AlloP may involve mechanisms beyond GABA_A_ receptor modulation. In addition to GABAergic functions, AlloP is found to promote autophagy in a murine Niemann-Pick Type C disease model (Liao et al., 2009) and in primary astrocyte cultures (Kim et al., 2012). These findings suggest that autophagy activation may exert neuroprotective effects (Liu et al., 2017). Indeed, we recently reported that in the retina AlloP activates autophagic flux and autophagy contributes to neuroprotection (Ishikawa et al., 2021).

**FIGURE 1:**
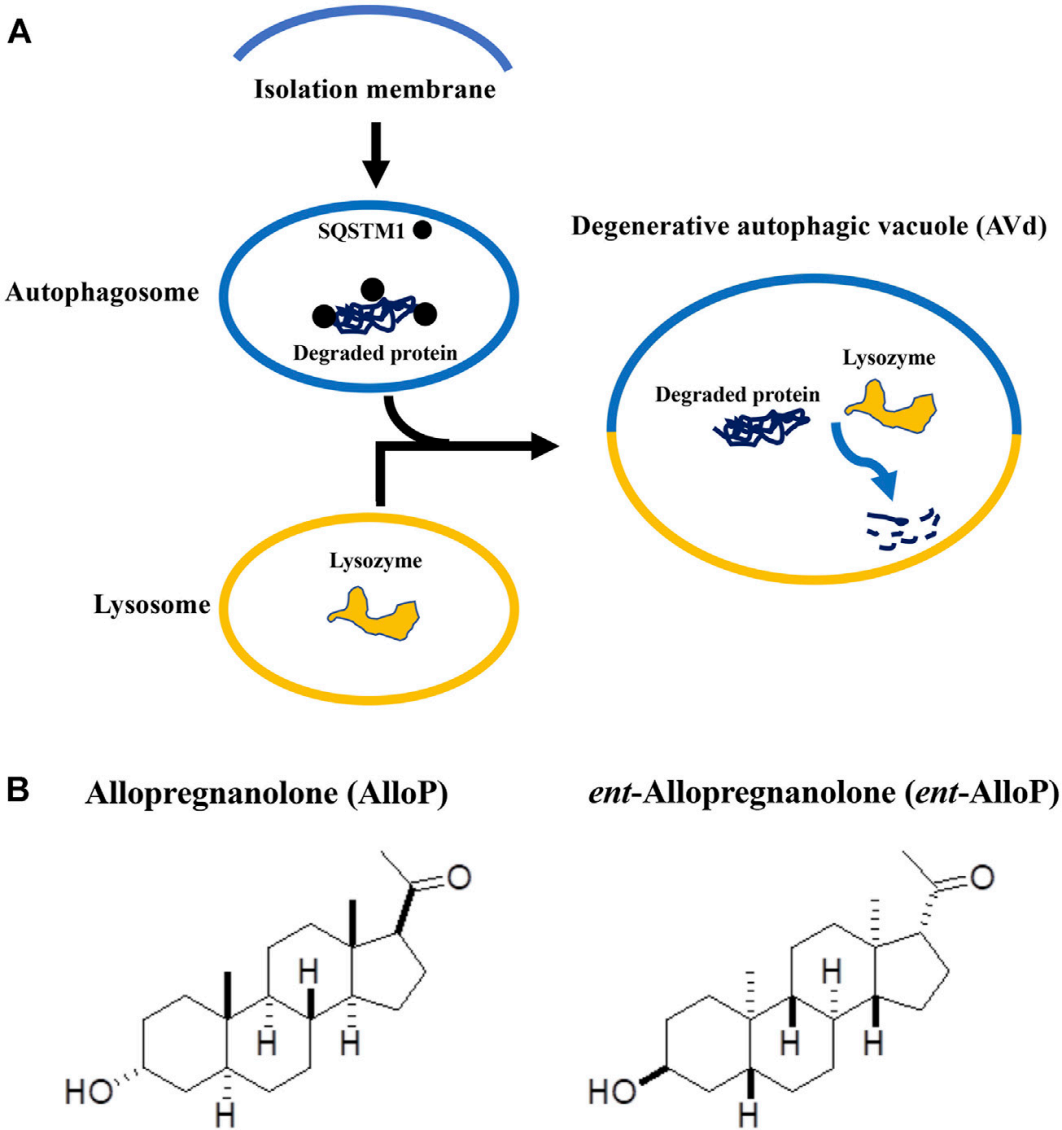
**(A)**. Key steps of autophagy flux. This figure is a modification of Figure 5 in the manuscript by Fujii et al. (2021). **(B)**. Structures of allopregnanolone (AlloP) and its enantiomer, *ent-*AlloP.

Autophagy is a highly conserved system that supplies nutrients to survive starvation (de Waal et al., 1986). Autophagy also responds to cellular stresses such as accumulation of damaged organelles or pathogens. When autophagy begins, cytoplasmic constituents are sequestered by an expanding double-membrane structure, called the isolation membrane (Takeshige et al., 1992). The isolation membrane completes sequestration, and results in formation of a double-membrane vesicle (autophagosome, AP) (Mizushima, 2007). APs then fuse with lysosomes to degrade their contents. These degrading structures are named degenerative autophagic vacuoles (AVds) or autolysosomes (Mizushima, 2007; Eskelinen, 2005; Hasegawa et al., 2015) (Figure 1A). Autophagy is considered to play an important role in pathogenesis of neurodegenerative diseases including glaucoma (Liton et al., 2009; Munemasa and Kitaoka, 2015; Russo et al., 2015).

Because GABA_A_ receptor-mediated actions might cause adverse effects in addition to neuroprotection, a compound similar to AlloP with selectivity for enhancing autophagic flux could be more desirable for therapeutic use. The synthetic enantiomer of AlloP (*ent*-AlloP) has weak actions on GABA_A_ receptor signaling (Wittmer et al., 1996; Hu et al., 1997, Figure 1B). In spite of its differences from AlloP, *ent*-AlloP may have neuroprotective effects in a murine model of Niemann-Pick Type C disease (Langmade et al., 2006), raising the possibility that *ent-*AlloP acts *via* mechanisms distinct from AlloP.

In the present study, we compared autophagic neuroprotection of *ent-*AlloP with AlloP using rat *ex vivo* and *in vivo* ocular hypertension (OHT) models.

## Materials and Methods

All procedures were approved by the Akita University Animal Studies Committee, and performed in accordance with the guidelines of the Policies on the Use of Animals and Humans in Neuroscience Research.

### Rat *ex vivo* Eyecup Preparation

28–32 day old male Sprague-Dawley rats were purchased from Charles River Laboratories International Inc. (Wilmington, MA) . The anterior half of enucleated eyes was removed, and the posterior eye cup was placed at the bottom of a 100 ml glass beaker filled with aCSF (artificial cerebrospinal fluid) containing (in mM): 124 NaCl, 5 KCl, 2 MgSO_4_, 2 CaCl_2_, 1.25 NaH2PO_4_, 22 NaHCO_3_, and 10 glucose, and incubated at 30°C for 24 h using a closed pressure-loading system (Ishikawa et al., 2014; 2021). A 95% O_2_-5% CO_2_ gas mixture was delivered into the incubation buffer.

In some experiments, AlloP (1 μM), *ent-*AlloP (1 μM), and 3-MA (10 mM) were administered in aCSF at the time of experiment, and incubated for 24 h. Eyecup preparations were preincubated with these drugs for 1 h at 30°C before pressure loading. After pressure loading at 10 mmHg or 75 mmHg for 24 h at 30°C, the pressure inside the chamber was gradually decreased.

### Rat *in vivo* OHT Model

8 week old male Sprague-Dawley rats were purchased from Charles River Laboratories. Anesthesia was induced with an intraperitoneal injection of a mixture of medetomidine hydrochloride (Cat #133-17474, CAS.No 86347-15-1, Wako Pure Chemical Industries Ltd., Osaka, Japan, 0.15 mg/kg), midazolam (Cat#135-13791, CAS.No 58786-99-5, Wako Pure Chemical Industries Ltd., 2 mg/kg), and butorphanol tartrate (Cat#021-19001, CAS.No 86347-15-1, Wako Pure Chemical Industries Ltd., 2.5 mg/kg). A 10 μl PBS solution of sterile 6 μm polystyrene microbeads (1 × 10^7^ microbeads/ml) (Molecular Probes, Eugene, OR, United States) was intracamerally injected using a single-step, sclero-corneal tunnel approach with a 35G-gauge nanoneedle (#LNAN-3505LM, Saito Medical Instruments Inc., Tokyo, Japan) connected to a 100 μl WPI Nanofil 100 microsyringe (World Precision Instruments, Inc., Sarasota, FL) as previously described (Ishikawa et al., 2021).

One week after microbead injection, rats were randomly divided into a non-treated OHT group, an AlloP injection group, and an *ent*-AlloP injection group. A total volume of 1 μl of 20% w/v (2-Hydroxypropyl)-β-cyclodextrin (2HBCD) saline solution containing AlloP or *ent-*AlloP (0.05%, w/v) was vitreally injected under halothane anesthesia using a Hamilton syringe adapted with a 35 gauge (G) nanoneedle 1 week after the beads were injected. Non-treated OHT rats received sterile 1 μl 20% 2HBCD with phosphate buffered saline (PBS) (vehicle control). As a further control, 1 μl 20% 2HBCD was injected into the vitrous chamber 1 week after intracameral injection with 10 μl of PBS.

OHT was monitored preoperatively, and at 3 days, 1 week, 2 weeks or 3 weeks after bead injection. IOP was measured with a rebound tonometer (TonoLab, Icare Finland, Vantaa, Finland). The eyes were excised for TUNEL staining, flat-mount preparation, electron microscopy, and Western Blot 3 weeks after beads were injected. Electroretinograms (ERGs) were also recorded 3 weeks after bead injection.

### Light Microscopy

After each experiment was complete, middle parts of retinal specimens were fixed in 1% paraform-aldehyde and 1.5% glutaraldehyde in 0.1 M phosphate buffer overnight at 4°C followed by post-fixation with 1% osmium tetroxide and 0.1 M phosphate buffer for 60 min as previously described (Ishikawa et al., 2014). Fixed retinas were dehydrated in graded ethanol, embedded in Epon 812 resin^TM^ (TAAB Laboratories, Aldermaston, United Kingdom) and cut into 1 μm thick sections. These semi-thin sections were stained with toluidine blue for light microscopy.

### Electron Microscopy

Ultrathin sections from retinal specimens embedded in epoxy resin were cut with a diamond knife and suspended over formvar-coated grids. After staining with uranyl acetate and lead citrate, ultrathin sections were viewed using a transmission electron microscope (H-7650, Hitachi High-Technologies Corp., Tokyo, Japan) according to previously described methods (Ishikawa et al., 2021). Numbers of AP and AVd inside nerve fiber layers (NFLs), ganglion cell layer (GCL), or axons were averaged based on the measurement of 10 different areas of 25 μm^2^ each from each sample (*n* = 10 for each condition). These morphometrical parameters were assessed by three raters who were unaware of the experimental conditions. Inter-examiner difference was not significant by one-way ANOVA followed by a *post-hoc* test as previously described. The density of AP and AVd are expressed as mean ± SEM, and evaluated by Tukey’s multiple comparison test compared to controls.

### Data Analysis of Morphometry

According to previously described methods (Ishikawa et al., 2014), the nerve fiber layer thickness (NFLT) was measured around 1,200 μm away from the center of the optic disc.

The density of degenerated cells in the GCL was calculated based on counting ten fields of 500 μm length (950–1,450 μm away from the center of the optic disc) in light micrographs (Ishikawa et al., 2014). Degenerated cells are characterized by nuclear chromatin clumping or necrosis.

The severity of neuronal damage was scored by light microscopy using a neuronal damage score (NDS). The NDS rates neuronal damage in the INL and the inner plexiform layer (IPL) on a 0-4 scale with 0 signifying no neuronal damage and 4 indicating very severe damage as described previously (Ishikawa et al., 2014, 2016).

The number of axons was calculated based on measurement of five different optic nerves from five eyes per experimental condition, and data are presented as axon density per 100 μm^2^.

NFLT, density of degenerated cells in the GCL, NDS, were calculated based on measurement of 10 different areas from five eyes per experimental condition by three raters who were unaware of the experimental conditions as described above (Ishikawa et al., 2021). NFLT, NDS, and the density of degenerated cells in the GCL were expressed as mean ± SD. Each data was compared to controls and evaluated by Tukey’s multiple comparison test.

### Preparation of Whole Mounted Retinas and Immunostaining

The retinas were detached from the pigment epithelium, flat-mounted, and fixed with 4% paraformaldehyde-0.1 M phosphate buffer overnight at 4°C. After rinse with PBS, retinas were immersed in 2% bovine serum albumin in PBS containing 0.5% Triton X-100, and incubated with rabbit anti-NeuN polyclonal antibody solution (Cat#ab104225, RRID:AB_10711153, Abcam, Cambridge, MA) (1:100) by gently shaking at 4°C, overnight. After rinsing 3 times using PBS, the retina was incubated in FITC-conjugated secondary antibody (goat anti-rabbit IgG (H&L) (Alexa Fluor^®^488) (Cat#ab150077, RRID:AB_2630356, abcam) (1:300). After rinsing 3 times with PBS, retinas were stretched on glass slides using 50% PBS and 50% glycerol. Retinal flat-mount preparations were imaged in each of the four defined retinal quadrants 4 mm from the optic nerve head using a confocal microscope according to previously described methods (Ishikawa et al., 2014, 2021). The density of NeuN positive RGCs per square millimeter was measured and compared in experimental retinas. NeuN positive RGCs were averaged the measurement of five different areas from five eyes per experimental condition.

### Apoptosis

According to previously described methods (Ishikawa et al., 2014, 2021), the cornea, lens and vitreous were surgically removed. The empty eye cup preparations were fixed with 4% paraformaldehyde-0.1 M phosphate buffer for 2 h at 4°C. These eyecup samples were embedded in optimal cutting temperature (O.C.T.) compound (Sakura Global Holdings, Tokyo, Japan), and rapidly frozen using liquid nitrogen. DeadEnd™ Fluorometric TUNEL System (Promega, Madison, WI) was used to detect the apoptotic cells according to the manufacturer’s instructions. Nuclei were counterstained with DAPI^TM^. The number of TUNEL positive cells in the GCL was normalized per 200 μm of retinal section.

### Western Blot Analysis

After homogenization of whole retinas, cellular proteins were extracted, separated by electrophoresis, transferred to nitrocellulose membranes and probed, as described previously (Ishikawa et al., 2021). Twenty micrograms of retinal extract were separated in SDS polyacrylamide gel electrophoresis using NuPAGE^TM^ 12% Bis-Tris Gel (Invitrogen, Carlsbad, CA) for microtubule-associated protein-1 light chain 3 beta (MAP1LC3B/LC3B) and NuPAGE^TM^ 4-12% Bis-Tris Gel (Invitrogen) for SQSTM1/p62 (sequestosome 1), and transferred to nitrocellulose membranes. The antibodies used for Western Blots were polyclonal rabbit anti-LC3B antibody (Cat# NB100-2220, RRID:AB_10003146, Novus Biologicals, Centennial, CO) and anti-SQSTM1 mouse monoclonal antibody (Cat#ab91526, RRID:AB_2050336, abcam). Immunoblots were developed using WesternBreeze^TM^ Chemiluminescent Immunodetection system (Invitrogen), and exposed to autoradiograph film (MXJB Plus^TM^; Kodak, Rochester, NY).

The density of Western Blot bands of the lipid-anchored form of LC3B (LC3B-II) and SQSTM1 was quantitatively analyzed using Image-Pro Plus software as previously described (Ishikawa et al., 2021). Four independent experiments were performed for each condition.

### Chemicals

AlloP was purchased from Wako Pure Chemical Industries, Ltd. (Cat#596-30841, CAS.NO 516-54-1; Osaka, Japan), and *ent-*AlloP (Wittmer et al., 1996) was synthesized by KK and DFC. All other chemicals were purchased from Sigma-Aldrich Corp. or Nacalai Tesque (Kyoto, Japan). AlloP and *ent-*AlloP were dissolved in DMSO as a 10 mM stock solution.

### Electroretinogram Recording

At 3 weeks after the induction of elevated IOP, the positive components of the scotopic threshold response (p-STR), an indicator of RGC function, were measured according to our previous report (Ishikawa et al., 2021). Briefly, rats were dark-adapted for 12 h and prepared for ERG recording under dim red light. Anesthesia was induced with an intraperitoneal injection of a mixture of medetomidine hydrochloride, midazolam, and butorphanol tartrate. Pupils were maximally dilated with topical 0.5% tropicamide (Santen Pharmaceuticals Co., Ltd., Osaka, Japan), and corneal anesthesia was achieved with a single drop of 0.4% oxybuprocaine hydrochloride (Santen Pharmaceuticals). Full-field ERGs were recorded simultaneously from both eyes using a PuRec^TM^ system (Mayo Co., Nagoya, Japan) as previously reported (Ishikawa et al., 2021).

### Statistics

All analyses were performed using a biomedical statistical computer program (http://www.gen-info.osaka-u.ac.jp/MEPHAS/dunnett.html). For multiple comparisons with the control and other conditions, we used Dunnett’s or Tukey’s multiple comparison test. For comparison of two groups, we used Wilcoxon Rank-Sum Test.

## Results

Effects of *ent-*AlloP on pressure-mediated retinal degeneration in an *ex vivo* model

**FIGURE 2:**
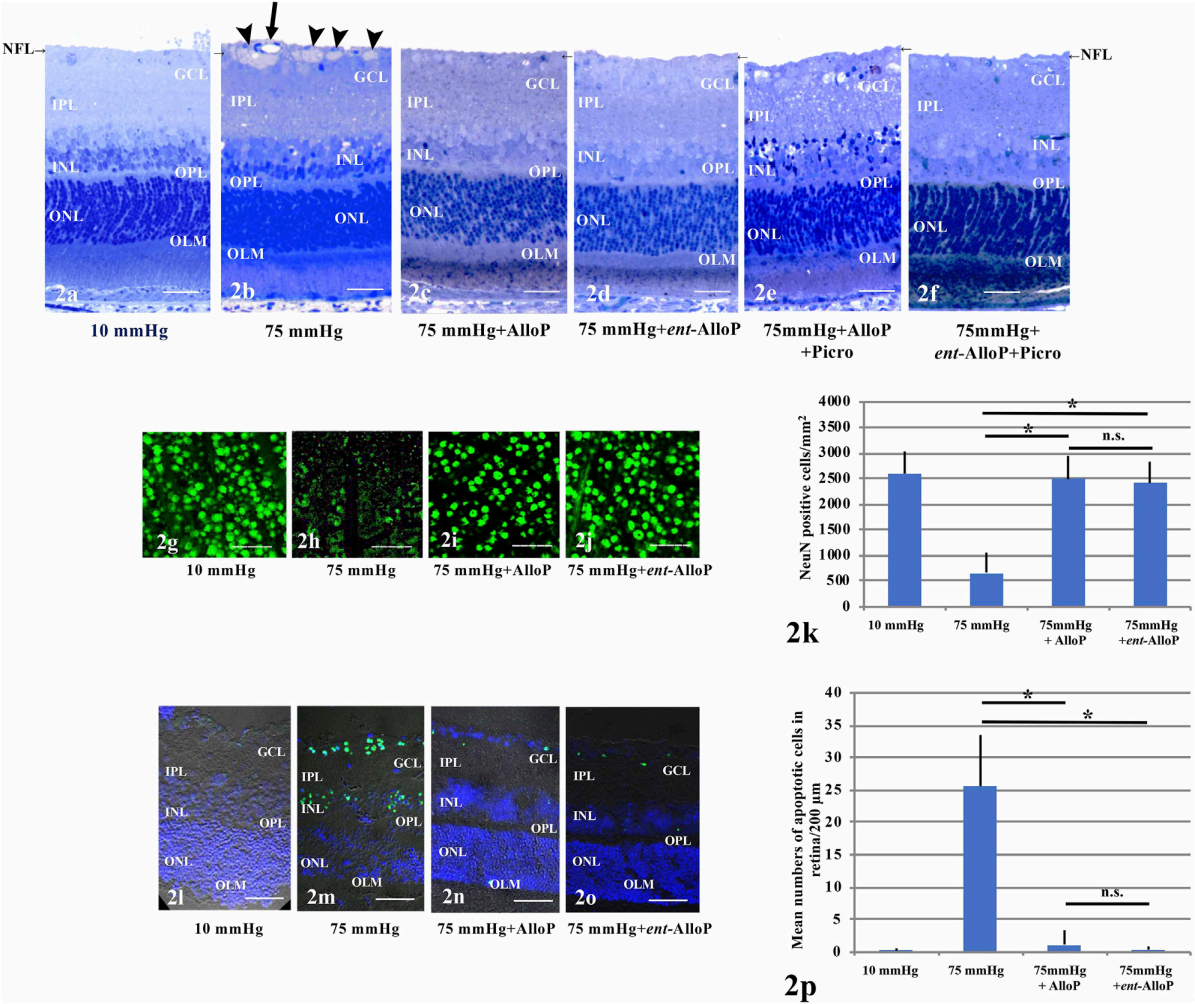
**(A–F)** Light micrographs of pressure-loaded retina. NFL is indicated with a short arrow. **(A)**. 10 mmHg.**(B)**. 75 mmHg. Arrowheads; axonal swelling. Arrow; blood vessel. **(C)**. Administration of 1 μM AlloP at 75 mmHg.**(D)**. Administration of 1 μM *ent-*AlloP at 75 mmHg. **(E)**. Administration of 1 μM AlloP and 1 μM picrotoxin. **(F)**. Administration of 1 μM *ent-*AlloP and 1 μM picrotoxin. Scale bars, 20 mm**(G–J)**. RGC survival and neuroprotection with AlloP and *ent-*AlloP in pressure-loaded whole mounted retinas. **(G)**. 10 mmHg.**(H)**. 75 mmHg. **(I)**. Administration of 1 μM AlloP at 75 mmHg. **(J)**. Administration of 1 μM *ent-*AlloP at 75 mmHg. Scale bars, 200 μm **(K)**. The number of immunopositive cells for anti-NeuN antibody in whole mount retinas (Tukey *p* < 0.05). **(L–O)**. TUNEL staining. **(L)**. 10 mmHg.**(M)**. 75 mmHg. Administration of 1 μM AlloP **(N)**. or 1 μM *ent-*AlloP **(O)**. significantly suppressed the number of TUNEL-positive cells at 75 mmHg. Scale bars, 30 μm **p.** Quantitative analysis of the number of apoptotic RGCs per 200 μm of retina section (Tukey *p* < 0.05).

Retinas exhibited normal appearance at 10 mmHg for 24 h incubation (Figure 2A) but showed axonal swelling in the nerve fiber layer (NFL) at 75 mmHg (Figure 2B). As we recently reported (Ishikawa et al., 2021), this damage is attenuated by 1 µM AlloP (Figure 2C). In addition to AlloP, we examined its enantiomer and found that 1 µM *ent-*AlloP also inhibited axonal swelling (Figure 2D). To determine the involvement of GABA_A_ receptors in neuroprotective effects of *ent-*AlloP, we administered 1 μM picrotoxin (PTX), a GABA_A_ receptor antagonist. As previously observed (Ishikawa et al., 2014, 2021), PTX dampened the neuroprotective effect of AlloP under hyperbaric conditions (Figure 2E). However, PTX did not alter the effects of *ent-*AlloP (Figure 2F), indicating that the mechanism underlying the neuroprotection of *ent*-AlloP is distinct from GABA_A_ receptor activation. Structural changes induced by pressure elevation and administration of neurosteroids or picrotoxin are analyzed in Table 1.

**TABLE 1:** Morphological changes of retinas after pressure-loading and neurosteroids treatment.

Condition	NFLT vs. RT (%) (*p value vs. 75 mmHg*)	NDS (*p*]	Damaged cells in GCL (*p*]
10 mmHg	1.3 ± 0.7 (Reference)	0.2 ± 0.4 (Reference)	1.3 ± 1.5 (Reference)
75 mmHg	11.3 ± 1.9 [-]	0.7 ± 0.5 [-]	16.2 ± 5.3 [-]
75 mmHg + 1 μM AlloP	1.7 ± 0.7 (*p < 0.05)	0.2 ± 0.4 (*p* > 0.05)	2.0 ± 1.7 (*p < 0.05)
75 mmHg + 1 μM *ent-*AlloP	1.6 ± 0.6 (*p < 0.05)	0.3 ± 0.5 (*p* > 0.05)	1.9 ± 1.2 (*p < 0.05)
75 mmHg + 1 μM AlloP + 1 μM Picro	3.8 ± 2.6 (*p < 0.05)	3.0 ± 0.8 (*p < 0.05)	23.9 ± 6.6 (*p < 0.05)
75 mmHg + 1 μM *ent-*AlloP + 1 μM Picro	1.5 ± 0.6 (*p < 0.05)	0.3 ± 0.5 (*p* > 0.05)	2.1 ± 1.1 (*p < 0.05)

Values are expressed as mean ± SD. NFLT vs. retinal thickness (RT) (%) means the NFLT percentage of total RT. Number of degenerated cells in the GCL was counted per 250 μm of retina. Statistical significance in each parameter were calculated using Dunnett’s test.

### *ent-*AlloP Preserves Neuronal Nuclear Antigen Under High Pressure

In whole mounted retinas, pressure elevation decreased cells positive for NeuN. Figure 2G demonstrates confocal images of NeuN-labeled RGCs of a control eye incubated at 10 mmHg. As previously reported (Ishikawa et al., 2021) the reduction of positive cells for NeuN by pressure elevation (75 mmHg) (Figure 2H) was attenuated by AlloP (Figure 2I). Similar to the effects of AlloP, *ent-*AlloP also preserved NeuN staining in the hyperbaric condition (Figure 2J). Figure 2K shows density of NeuN positive RGCs in the retina in each condition.

### *ent-*AlloP Prevents Pressure-induced Apoptosis

As previously reported (Ishikawa et al., 2016, 2021), in the GCL and outer nuclear layer (ONL) TUNEL-positive cells are infrequently observed at 10 mmHg (Figure 2L) but are apparent at 75 mmHg (Figure 2M). Similar to AlloP (Figure 2N), we observed *ent-*AlloP prevented apoptosis at 75 mmHg (Figure 2O). Figure 2P shows the number of TUNEL-positive cells in the GCL in each condition.

### Autophagy Vacuoles Induced by High Pressure and Effects of *ent*-AlloP

**FIGURE 3:**
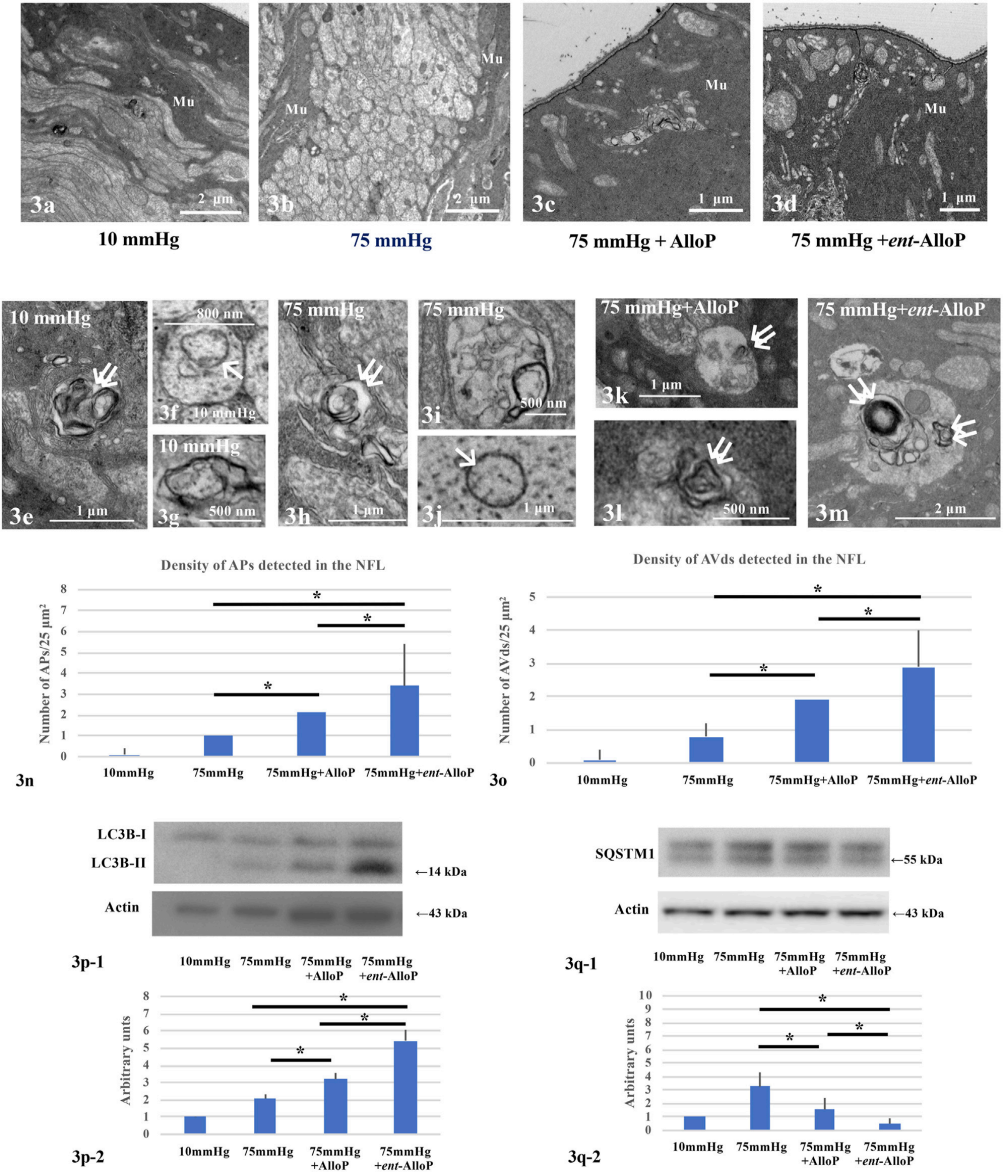
Electron micrographic **(A–O)** and Western Blot analyses **(P,Q)** of *ex vivo* retinas. **(A–D)** Low magnification of the NFL at 10 mmHg **(A)**, 75 mmHg **(B)**, 75 mmHg with AlloP administration **(C)**, and 75 mmHg with *ent*-AlloP administration **(D)**. Mu, Müller cell. **(E–G)** AVds (double arrows in **e**) and AP (single arrow in **F**) in the NFL at 10 mmHg.**(H–J)** AVds (double arrows in **H**) and AP (single arrow in **J**) in the NFL at 75 mmHg. **(K,L)** Administration of AlloP (1 μM) at 75 mmHg. AVds are indicated with double arrows. **(M)** Administration of *ent*-AlloP (1 μM) at 75 mmHg. Double arrows, AVd. **(N)** The density of AP per 50 μm^2^ of retina. (Tukey *p* < 0.05). **(O)** The density of AVd per 50 μm^2^ of retina (Tukey *p* < 0.05). **P-1.** Representative immunoblotting for LC3B and actin. **P-2.** Relative densitometry analysis of LC3B-II expression (n = 4 per experiment, Tukey *p* < 0.05). **Q-1.** Representative immunoblotting for SQSTM1 and actin. **Q-2.** Relative densitometry analysis of SQSTM1 expression (*n* = 4 per experiment, Tukey *p* < 0.05).

We have observed that in the NFL AlloP robustly increases autophagosomes (APs) whereas APs are minimal at control pressure (10 mmHg, Figures 3A,E–G) or elevated pressure (70 mmHg) without AlloP (Figures 3B,H–J). We also observed that AlloP significantly increases degenerative autophagic vacuoles (AVds) (Figures 3C,K,L) as reported previously (Ishikawa et al., 2021), whereas AVds are minimal at control pressure (10 mmHg) or elevated pressure (75 mmHg) without AlloP. We also found that *ent*-AlloP increased APs and AVds (Figures 3D, M). These observations suggest the possibility that *ent*-AlloP is more efficient in promoting autophagy flow compared to AlloP (Figures 3N,O). Therefore, we next examined how *ent*-AlloP affects autophagy markers at elevated pressure.

### Autophagy Markers and Effects of *ent*-AlloP on Autophagy Flow

LC3B protein plays a critical role in autophagy, and the lipidated form of LC3B (LC3B-II), an indicator of autophagosome formation, displayed a band at approximately 14 kDa. Quantitative Western Blot analysis demonstrated that administration of both neurosteroids significantly increased LC3B-II expression compared to drug-free pressure elevation (Figure 3P–1). However, the increase is more robust with *ent*-AlloP, indicating again that *ent*-AlloP is a more efficient enhancer of autophagy than AlloP (Figure 3P-2).

We next examined expressional changes of SQSTM1, a widely used predictor of autophagic flux that is incorporated into mature autophagosomes and accumulates when autophagic flux is inhibited (Mizushima and Yoshimori, 2007). Western Blotting revealed double bands at approximately 57 and 55 kDa using SQSTM1 antibodies (Figure 3Q-1). Our previous study (Ishikawa et al., 2021) revealed that the 55 kDa band is specific for SQSTM1. Although we have shown that increased expression of SQSTM1 by elevated pressure was partially dampened by AlloP (Ishikawa et al., 2021), *ent*-AlloP more effectively depressed SQSTM1 than AlloP (Figures 3Q-1, 3Q-2).

### 3-MA Blocks Autophagy and Inhibits Neuroprotective Effects of *Ent*-AlloP

**FIGURE 4:**
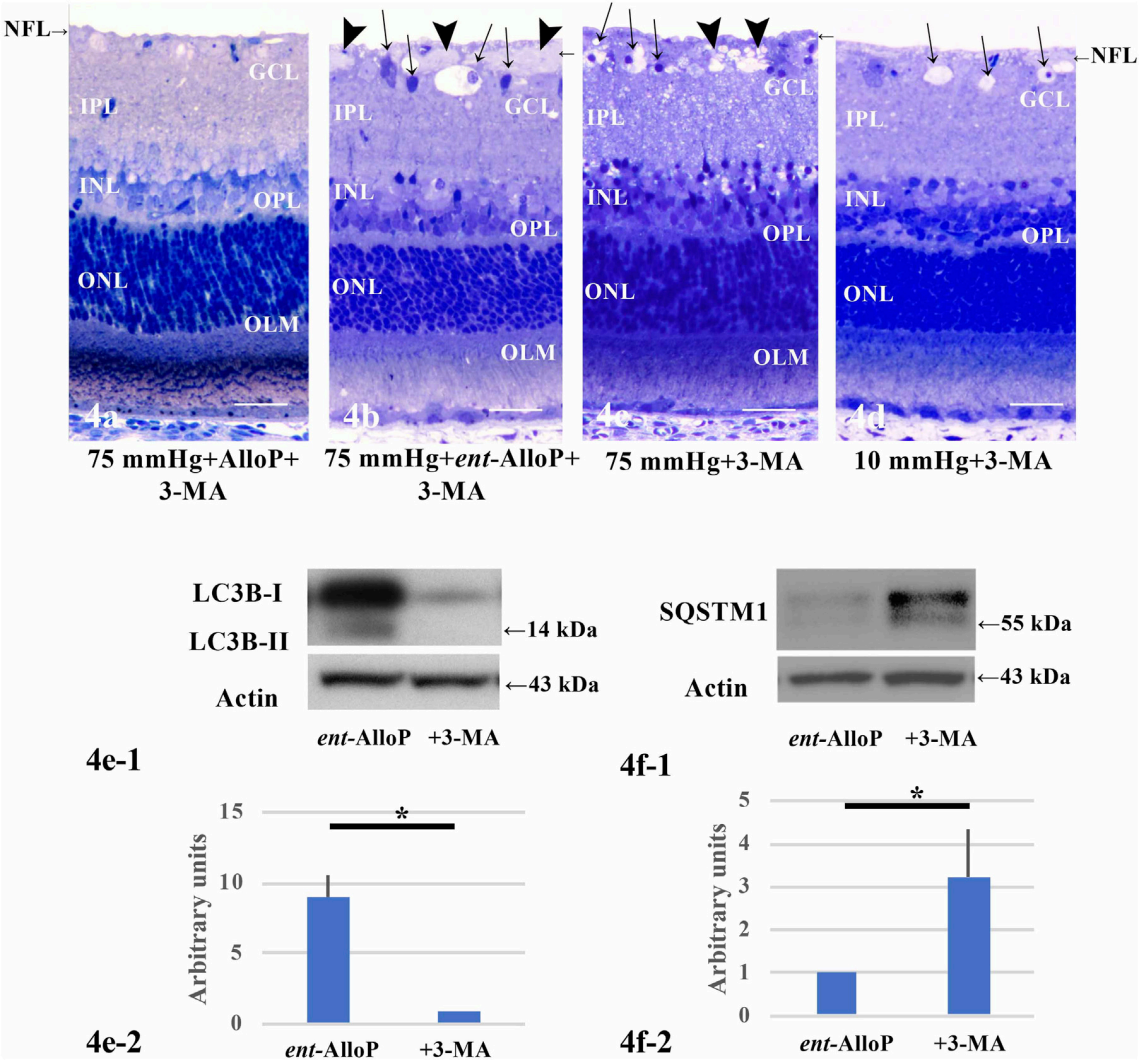
**(A–D)** Light micrographs showing effects of pressure elevation, neurosteroids, and 3-MA on retinal morphology. NFL is indicated with a short arrow. **(A)** Administration of 10 mM 3-MA showed no differences in the retina incubated with 1 μM AlloP at 75 mmHg.**(B)** Administration of 10 mM 3-MA dampened protective effects of 1 μM *ent-*AlloP at 75 mmHg. Arrowheads; axonal swelling. Arrows; RGC degeneration. **(C)** 3-MA (10 mM) alone induced severe damage at 75 mmHg. Arrowheads; axonal swelling. Arrows; RGC degeneration. **(D)** 3-MA (10 mM) also induced degeneration at 10 mmHg. Arrows; RGC degeneration or vacuoles in GCL. Scale bars, 20 μm. **E-1.** Representative Western Blot analyses of LC3B proteins in pressure-loaded retinas (75 mmHg) treated with 1 μM *ent-*AlloP alone or with 1 μM *ent-*AlloP and 10 mM 3-MA. **E-2.** Relative densitometry analysis of LC3B-II expression in the retina incubated with *ent-*AlloP or with 1 μM *ent-*AlloP and 10 mM 3-MA at 75 mmHg (*n* = 4 per experiment, Wilcoxon Rank-Sum Test *p* < 0.05). **F-1.** Representative Western Blot analyses of SQSTM1 proteins in pressure-loaded retinas (75 mmHg) treated with 1 μM *ent-*AlloP alone or with 1 μM *ent-*AlloP and 10 mM 3-MA. **F-2.** Relative densitometry analysis of SQSTM1 expression. Administration of 10 mM 3-MA significantly increased SQSTM1 expression in the retina incubated with *ent-*AlloP at 75 mmHg (*n* = 4 per experiment, Wilcoxon Rank-Sum Test, *p* < 0.05).

To determine whether autophagy plays a key role in retinal protection by *ent*-AlloP, we examined 3-methyladenine (3-MA), an inhibitor of autophagic flux. At 75 mmHg, 3-MA did not alter neuroprotection by AlloP (Figure 4A), but dampened the effects of *ent*-AlloP (Figure 4B). We also found that 3-MA alone was neurodegenerative at both 75 mmHg (Figure 4C) and 10 mmHg (Figure 4D), indicating that autophagy is important for maintaining retinal integrity even under control conditions. A combination of *ent*-AlloP and 3-MA significantly increased damaged cells in the GCL compared to controls at 75 mmHg (*p* < 0.05) and overcame the protective effects of *ent*-AlloP at high pressure. Structural changes induced by pressure elevation and administration of neurosteroids or 3-MA are analyzed in Table 2.

**TABLE 2:** Morphological changes of retinas after 3-MA and neurosteroids treatment.

Condition	NFLT vs. RT (%) (*p value vs. 75 mmHg*)	NDS (*p*)	Damaged cells in GCL (*p*)
75 mmHg + 10 mM 3-MA	1.4 ± 1.0 (Reference)	1.4 ± 0.5 (Reference)	17.7 ± 3.4 (Reference)
75 mmHg + 1 μM AlloP + 10 mM 3-MA	1.8 ± 0.4 (*p* > 0.05)	0.3 ± 0.5 (*p < 0.05)	4.9 ± 2.1 (*p < 0.05)
75 mmHg + 1 μM *ent-*AlloP + 10 mM 3-MA	11.4 ± 0.8 (*p < 0.05)	1.4 ± 0.5 (*p* > 0.05)	17.1 ± 6.0 (*p* > 0.05)

Values are expressed as mean ± SD. NFLT vs. retinal thickness (RT) (%) means the NFLT percentage of total RT. Number of degenerated cells in the GCL was counted per 250 μm of retina. Statistical significance in each parameter were calculated using Dunnett’s test.

We also examined whether 3-MA altered the effects of *ent*-AlloP on autophagic markers, and found that upregulation of LC3B-II levels induced by *ent-*AlloP was dampened by 3-MA (Figures 4E-1, 4E-2). Also, the decrease of SQSTM1 levels induced by *ent*-AlloP at 75 mmHg was reversed by 3-MA (Figures 4F–1, 4F-2). These findings indicate that *ent-*AlloP likely acts by enhancing autophagic flow.

### Rat *in vivo* OHT Model Induced by Intracameral Injection of Microbeads

**FIGURE 5:**
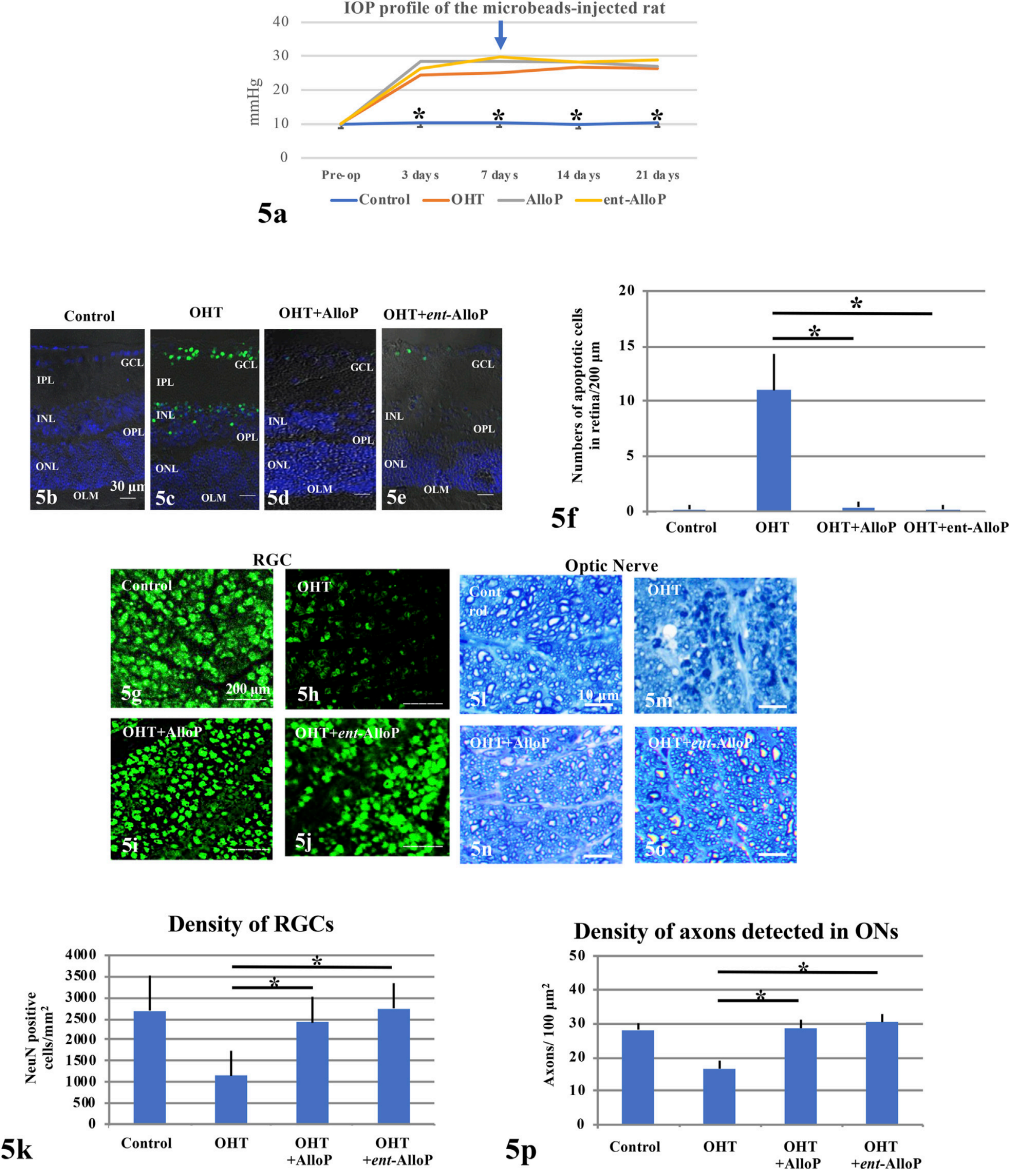
Effects of IOP and neurosteroids on RGC or axonal survival. **(A)** IOP profiles. An arrow marks the day when drugs were injected (day 7). **(B–E)** TUNEL staining. **(B)** Control eye. **c.** Non-treated OHT eye. **(D,E)** Administration of 1 μM AlloP **(D)** or 1 μM *ent-*AlloP **(E)** in OHT eyes. Scale bars, 30 μm. **(F)** The number of TUNEL-positive RGCs per 200 μm of retinal sections (Tukey *p* < 0.05). **(G–J)** Confocal images of NeuN-labeled RGCs. **(G)** Control eye. **(H)** Microbead-injected OHT eye without neurosteroid administration. **(I,J)** Administration of 1 μM AlloP **(I)** or 1 μM *ent-*AlloP **(J)**. Scale bars, 200 μm. **(K)** The density of immunopositive cells for NeuN antibody in the whole mount retina under each condition (Tukey *p* < 0.05). **(L–O)** Light micrographs of the optic nerves 3 weeks after microbead injection. **(L)** Control eye. **(M)** OHT. **(N,O)** Administration of 1 μM AlloP **(N)** or 1 μM *ent-*AlloP **(O)**. Scale bars, 10 μm. **(P)** Effects of IOP elevation, AlloP and *ent-*AlloP on Axon number in whole mount retinas (Tukey *p* < 0.05).

Ocular hypertension (OHT) was induced by injection of sterile 6 μm polystyrene microbeads into the anterior chamber. AlloP or *ent*-AlloP was administered as a one-time intravitreal injection 1 week following bead injection. Three weeks after bead injections, IOP increased to 26.1 ± 3.1 mmHg in non-treated OHT eyes compared to 10.4 ± 1.0 mmHg in control eyes, whereas it was 27.1 ± 3.1 mmHg in eyes treated with AlloP, similar to what we reported previously (Ishikawa et al., 2021). Like AlloP, we observed that *ent-*AlloP did not depress IOP (28.9 ± 5.3 mmHg, Figure 5A).

In spite of sustained elevation of IOP, both neurosteroids are effective neuroprotectants. Apoptosis activation as detected as TUNEL-positive cells in the GCL was clearly suppressed by *ent*-AlloP and the efficacy was comparable with AlloP (Figures 5B–F). Furthermore, the reduction of retinal ganglion cells (RGSs) by IOP elevation was prevented by *ent*-AlloP and the efficacy was comparable to AlloP (Figures 5G–K). Moreover, axonal loss in the optic nerve by IOP elevation was well prevented by both neurosteroids (Figures 5L–P).

### Autophagy Vacuoles and Markers by IOP Elevation and Effects of *ent-*AlloP

**FIGURE 6:**
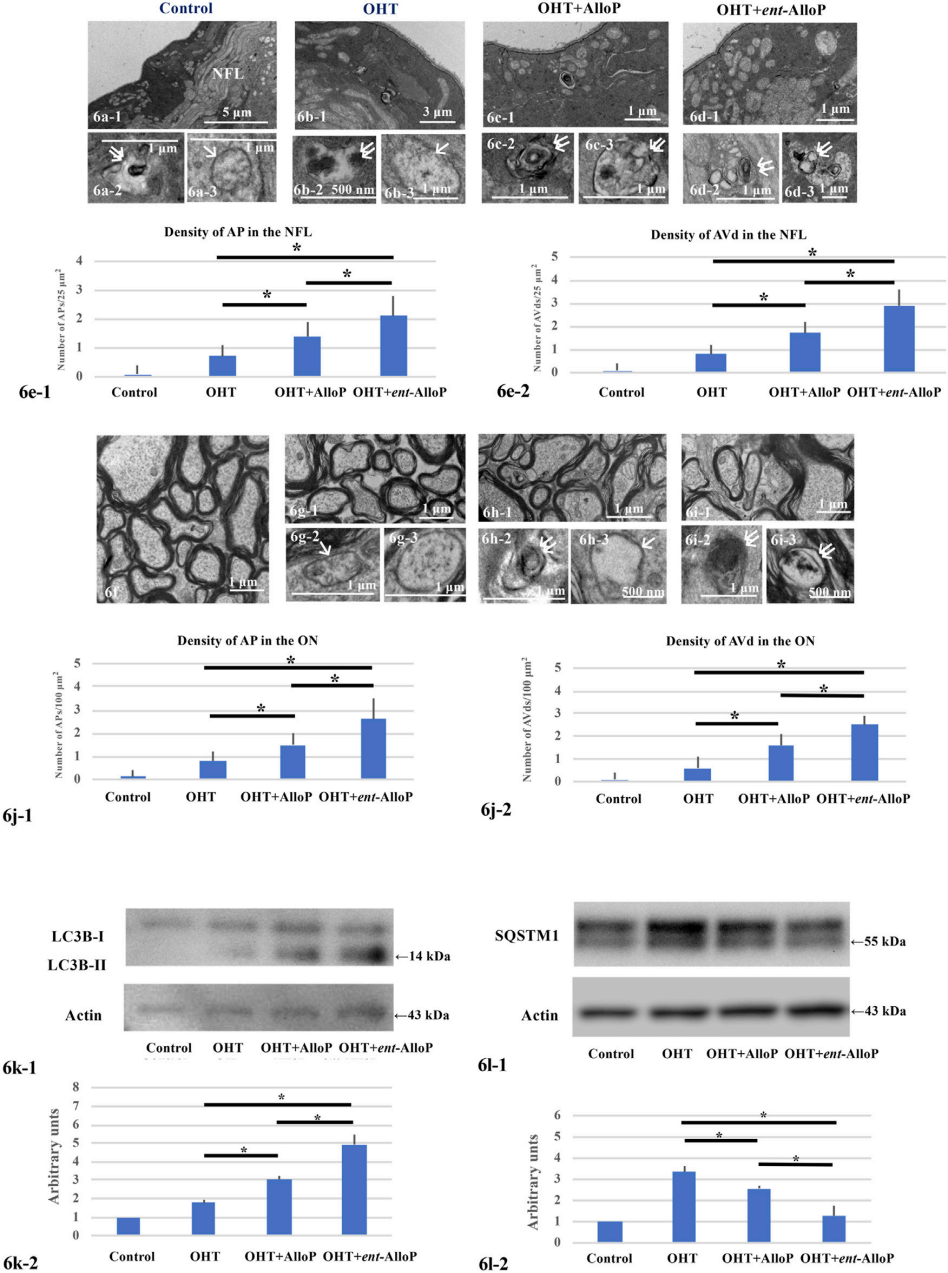
Electron micrographs and Western Blotting in *in vivo* OHT eyes. **(A–D)** Electron micrographs of the NFL. **(A-1,A-2,A-3)**. Control eyes. NFL, nerve fiber layer. **A-1.** Low magnification. **(A-1,A-3)**. AVd (double arrows in **A-2**) and AP (single arrow in **A-3**). **B-1, B-2, B-3.** Non-treated OHT eye. **B-1.** Low magnification. **B-2, B-3.** AVd **(B-2)** indicated by double arrows and AP **(B-3)** indicated by single arrow. **C-1, C-2, C-3.** OHT eyes treated with AlloP. **C-1.** Low magnification. **C-2, C-3.** AVds indicated by double arrows. **D-1, D-2, D3**. OHT eyes treated with *ent-*AlloP. **d-1.** Low magnification. **D-2, D-3.** AVds (double arrows). **E-1 and E-2.** The density of APs **(e-1)** and AVds **(E-2)** (*n* = 10 per experiment, Tukey *p* < 0.05). **f-i.** Electron micrographs of optic nerves. **f.** Control eyes. **G-1, G-2, G-3.** OHT eye. **G-1.** Low magnification. **g-2.** AP (arrow in **G-2**). **G-3.** AP. **H-1, H-2, H-3.** OHT eye treated with AlloP. **H-1.** Low magnification. **H-2.** AVd (double arrows). **H-3.** AP (single arrow). **I-1, I-2, I-3.** OHT eye treated with *ent-*AlloP. **I-1.** Low magnification. **I-2 and I-3.** AVds (double arrows). **J**. The density of APs **(J-1)** and AVds **(J-2)** (Tukey *p* < 0.05). **(K, L)** Western Blot Analysis. **K-1.** Representative immunoblotting for LC3B and actin. **K-2.** Relative densitometry analysis of LC3B-II expression (*n* = 4 per experiment, Tukey *p* < 0.05). **L-1.** Representative immunoblotting for SQSTM1 and actin. **L-2.** Relative densitometry analysis of SQSTM1 expression (*n* = 4 per experiment, Tukey *p* < 0.05).

Effects of neurosteroids on changes in autophagy vacuoles and markers following IOP elevation are similar to those observed in the *ex vivo* model. Consistent with our previous study (Ishikawa et al., 2021), APs and AVds are minimal in control eyes (Figures 6A-1, A-3) or OHT eyes (Figures 6B-1, B-2, B-3). AlloP increased APs and AVds in the NFL of OHT eyes (Figures 6C-1, C-2, C-3). Similarly, *ent*-AlloP increased the numbers of APs and AVds 3 weeks after IOP elevation in the same areas (Figures 6D-1, D-2, D-3 ). Number of APs and AVd are summarized in Figures 6E-1, E-2, with greater effects of *ent*-AlloP compared to AlloP. AlloP and *ent*-AlloP also induced similar changes in the optic nerves, again with greater effects of *ent*-AlloP (Figures 6F–J).

We have shown that AlloP increases LC3B-II expression compared to drug-free pressure elevation (Figures 6K-1, K-2). Now we found that the increase was more robust with *ent*-AlloP, indicating again that *ent*-AlloP is a more efficient accelerator of autophagy than AlloP.

Increased expression of SQSTM1 by elevated pressure was partially dampened by AlloP as we showed previously (Ishikawa et al., 2021); again, *ent*-AlloP more effectively depressed SQSTM1 than AlloP (Figures 6L-1, L-2).

### Intravitreal Neurosteroid Injection Preserves Scotopic Threshold Responses in Rat *in vivo* OHT Model.

**FIGURE 7:**
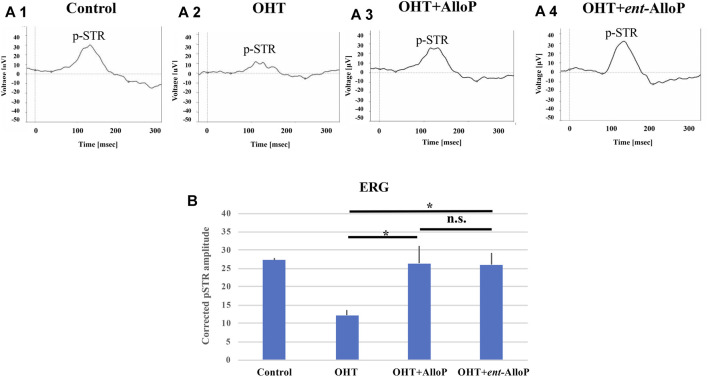
ERG analyses in *in vivo* OHT eyes. **A-1** to **A-4**. Representative ERG tracing in each experimental condition 3 weeks after intracameral bead injection. **A-1.** Control. **A-2.** OHT. **A-3.** Intravitreal administration of AlloP. **A-4.** Intravitreal administration of *ent-*AlloP. **(B)** Comparison of corrected p-STR amplitudes obtained from each experimental condition (*n* = 4 per each experiment, Tukey *p* < 0.05).

3 weeks after bead injections, the positive amplitude of the p-STR was significantly decreased in OHT eyes compared to control eyes (Figures 7A-1, A-2). A single intravitreal injection of AlloP and *ent-*AlloP prevented the decrease in p-STR amplitude with similar effects of the two neurosteroids (Figures 7A-3, A-4). A quantitative measurement of corrected p-STR is summarized in Figure 7B.

## Discussion

Using *ex vivo* and *in vivo* OHT models, we recently reported that AlloP enhances autophagy flow and found that this enhancement results in neuroprotection (Ishikawa et al., 2021). Another mechanism underlying AlloP neuroprotection is GABA_A_ receptor potentiation (Ishikawa et al., 2014). For AlloP, enhancement of autophagy flow and potentiation of GABA_A_ receptors may have independent and perhaps synergistic effects for neuroprotection (Figure 4). However, the activation of GABA_A_ receptors seems essential for AlloP to protect the retina because PTX totally voids the neuroprotective effects (Figure 2E). Even if the therapeutic potential of AlloP expected in glaucoma and other pathologic conditions is mediated by GABA_A_ receptors, activation of GABA_A_ receptors could be a drawback because of adverse effects such as sedation, confusion, weakness, and perhaps changes in vision. Such adverse effects are minimized in the unnatural enantiomer, which does not significantly potentiate GABA_A_ receptors. AlloP and *ent*-AlloP are mirror image molecules that have markedly different effects on GABA_A_ receptors (Wittmer et al., 1996; Covey, 2009) (Figure 1B), yet behave similarly in hydrophobic environments including cell membranes (Alakoskela et al., 2007). If *ent*-AlloP protects retinas only via autophagy enhancement, it could be more useful therapeutically than AlloP itself.

In this study, we made several important observations. First, we found that *ent*-AlloP protects the retina from high pressure damage in both *ex vivo* and *in vivo* models. Of importance is that the neuroprotection of *ent*-AlloP matches that of AlloP, supporting its potential as a therapeutic approach. Second, different from AlloP, however, neuroprotection by *ent*-AlloP in the *ex vivo* system was still observed in the presence of PTX, indicating that the mechanism underlying the neuroprotection is, as expected, independent of GABA_A_ receptors. This finding also implies that *ent*-AlloP could be more neuroprotective than AlloP, because it is expected that the neuroprotection remains even under conditions of ‘disinhibition’, which may cause various neuronal problems (Knutson et al., 2015).

Third, the present data demonstrate that the therapeutic potential of *ent*-AlloP involves autophagy enhancement. While apoptosis kills RGCs in glaucoma (Guo et al., 2005; Kato et al., 2012), autophagy may serve either a neurotoxic or neuroprotective role (Spalding et al., 2005) and is linked with apoptosis (Wang et al., 2015). Indeed, autophagy often coexists with apoptosis in other rat glaucoma models. After IOP elevation by episcleral vein cauterization in rats, LC3B-II and the LC3B-II/I ratio in RGCs have been reported to increase (Park et al., 2012). LC3B-II increase has also been shown in a rat ischemic reperfusion model (Piras et al., 2011), laser photocoagulation model (Kitaoka et al., 2013), and optic nerve transection model (Rodríguez-Muela et al., 2012). Because LC3B-II correlated with autophagosome formation (Kabeya et al., 2000), the increase in LC3B-II or the LC3-IIB/I ratio might indicate induction of autophagy. Autophagic vacuoles were also detected in these models (Piras et al., 2011; Rodríguez-Muela et al., 2012; Kitaoka et al., 2013; Oku et al., 2019). Similarly, autophagy in RGCs has been observed in a rhesus monkey OHT glaucoma model (Deng et al., 2013). Consistent with autophagy induction in these reports, we have shown LC3B-II induction (Figures 2P, 5K) and increases in APs and AVds in both the *in vivo* and *ex vivo* models (Figures 2N, 2O, 5J).

Given that autophagy is observed in glaucoma models, it is important to consider whether autophagy is neuroprotective or neurodegenerative. We addressed this issue using 3-MA, an agent that blocks autophagy by inhibiting class III phosphoinositide 3-kinase (PI3K), an enzyme required for membrane dynamics in autophagic vesicle trafficking and autophagosome formation (Seglen and Gordon, 1982).

Similar to a model of dementia, in which inhibition of early autophagy with 3-MA is neuroprotective (Lee and Gao, 2009), 3-MA reduces apoptosis in the ischemic reperfusion model (Piras et al., 2011), suggesting that targeting autophagy could be a treatment for glaucoma. A similar interpretation is shown in an optic nerve crush model (Koch et al., 2010). Furthermore, 3-MA, which attenuates LC3B expression after episcleral vein cauterization, reduces apoptosis in this glaucoma model (Park et al., 2018). Taken together, these reports suggest that autophagy tends to be neurodegenerative in glaucomatous eyes. In contrast to these reports, however, we observed 3-MA-induced degeneration of RGC at both low and high pressures (Figures 3C,D). Consistent with our findings, 3-MA is neurodegenerative even in control retinas (Rodríguez-Muela et al., 2012). Moreover, in a laser photocoagulation glaucoma model, axon numbers are further decreased by 3-MA (Kitaoka et al., 2013). Additionally, rapamycin, an agent that activates autophagy, rescues RGCs in a rat episcleral vein cauterization model (Su et al., 2014), in an optic nerve transection model (Rodríguez-Muela et al., 2012) and in an ischemic reperfusion model (Russo et al., 2018). Even if the role of autophagy in glaucoma is not fully settled, our observations, along with these latter reports, suggest that inhibition of autophagy flow could be deleterious and that pharmacological autophagy enhancement is neuroprotective in glaucoma.

We have reported that AlloP enhances autophagy in our OHT models (Ishikawa et al., 2021). In both models, AlloP increases expression of LC3B-II. Interestingly, AlloP also increases APs and AVd. Because these changes were not seen in OHT alone, we concluded that AlloP is an enhancer of autophagy and the enhancement contributes to neuroprotection. Previously we found that high pressure induces AlloP production in the *ex vivo* OHT model (Ishikawa et al., 2014). The endogenous AlloP induced by high pressure alone, however, is not sufficient to evoke robust activation of autophagy and, therefore, is insufficient to prevent apoptosis. Supplemental exogenous AlloP could thus be a good therapeutic strategy but the further enhancement of GABA_A_ receptors may limit this application. In this sense, *ent*-AlloP could be a preferred way to accelerate autophagy more selectively.

To determine whether *ent*-AlloP induced neuroprotective autophagy, we examined the pharmacological effects of 3-MA on LC3B proteins. We found that 3-MA attenuated LC3B-II upregulation induced by *ent-*AlloP (Figure 3E). 3-MA also upregulated SQSTM1 levels in spite of the presence of *ent*-AlloP (Figure 3F). It is known that SQSTM1 levels inversely correlate with autophagy activity (Mizushima and Yoshimori, 2007), and SQSTM1 accumulates when autophagy is inhibited (Bjørkøy et al., 2009). In pathological conditions, there is a significantly high level of SQSTM1, leading to accumulation of damaged mitochondria and excessive ROS generation (Johansen and Lamark, 2011). Moreover, an increase in SQSTM1 with a decrease in the LC3B-II/I ratio is associated with an impairment of the autophagy process in the optic nerve crush model (Oku et al., 2019). Thus, reduction of SQSTM1 expression by *ent-*AlloP (Figures 2Q, 5L) and enhancement by 3-MA (Figure 3F) again support the effective activation of autophagy flow by *ent-*AlloP. Taken together, these findings indicate that *ent-*AlloP activates autophagy flow more effectively than AlloP. Because of this, *ent*-AlloP could be a preferred neuroprotectant even if it does not significantly potentiate GABA_A_ receptors.

Previously, in the *ex vivo* glaucoma model we showed that simultaneous administration of AlloP with the autophagy inhibitor bafilomycin A1 induced retinal degeneration at 75 mmHg (Ishikawa et al., 2021), indicating that autophagy plays a key role in AlloP-mediated neuroprotection. By contrast, the present study demonstrated that AlloP exerted substantial protection even in the presence of 3-MA. This discrepancy may be due to different pharmacological mechanisms of 3-MA and Bafilomycin A1. Bafilomycin A1 inhibits lysosomal degradation activity and blocks autophagosome-lysosome fusion. On the other hand, 3-MA inhibits initiation of autophagy by blocking class III PI3K. Additionally, 3-MA inhibits class I PI3K, and can induce cell survival through a serine/threonine kinase (Akt) and other kinases (Chen and Yao, 2021). Activated Akt plays a pivotal role in survival of neuronal cells via PI3K/Akt signaling pathway. In P19-N neurons, AlloP prevents NMDA-induced excitotoxicity by preserving PI3K/Akt signaling pathway (Xilouri and Papazafiri, 2008), suggesting that neuroprotection by AlloP in the presence of 3-MA may be mediated by preservation of PI3K/Akt signaling.

While we used 3-MA to inhibit autophagy, it is important to note that prior studies indicate that this agent has complex effects. Wu et al. (2010) reported that 3-MA suppresses starvation-induced autophagy in cultured mouse embryonic fibroblasts but paradoxically promotes autophagy in nutrient-rich conditions. Similar to nutrient deprivation, acute IOP elevation is a substantial cellular stress to neurons and induces mitochondrial dysfunction, resulting in energy deficiency (Ju et al., 2008). Thus, if retinal stress following IOP elevation is comparable to nutrient deficiency in cultured cells, it is likely that 3-MA was an effective autophagy inhibitor in our experiments.

We conclude that *ent*-AlloP and AlloP, may promote neuroprotection in glaucomatous damage but that *ent*-AlloP is more selective and effective in activating autophagy, possibly providing unique advantages in different clinical situations. These results also have implications for the development of neurosteroids as treatments for other neurodegenerative disorders. Future studies should determine the cellular mechanisms by which autophagy enhancement protects retinal neurons from glaucoma (Boya 2017).

## Data Availability

The original contributions presented in the study are included in the article/Supplementary Material, further inquiries can be directed to the corresponding author.
