# Supplementary figures and images for: The Enantiomer of Allopregnanolone Prevents Pressure-Mediated Retinal Degeneration Via Autophagy
Source: Front Pharmacol. 2022 Mar 16;13:855779. doi: 10.3389/fphar.2022.855779 (PMC8966700; doi:10.3389/fphar.2022.855779)

Fig. 4e-1

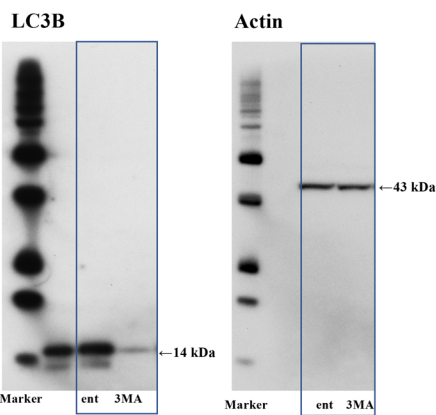

Fig. 4f-1

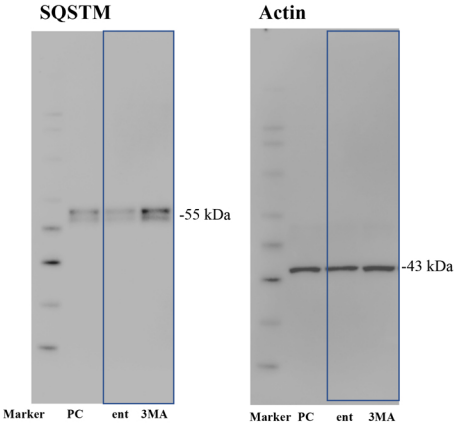

Supplement: Supplementary file 1 [file DataSheet2.PDF]

Source data of Western blotting

Fig. 6k-1

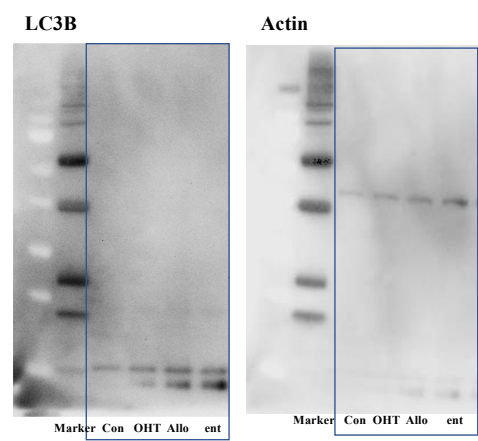

Fig. 6l-1

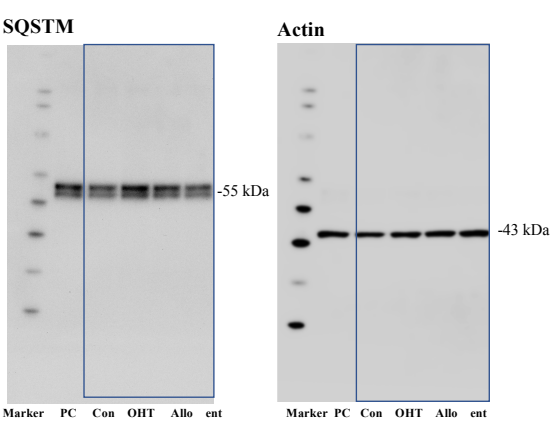

Supplement: Supplementary file 3 [file DataSheet3.PDF]

Source data of Western blotting

Fig. 3p-1

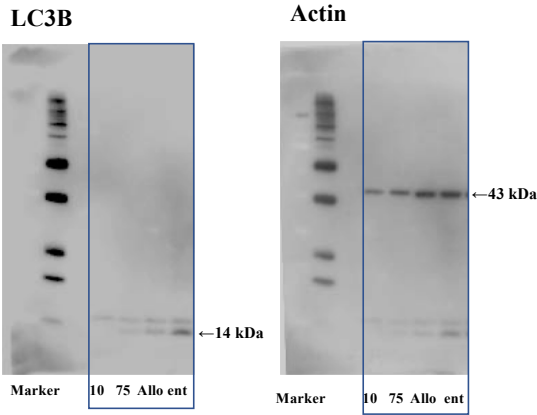

Fig. 3q-1

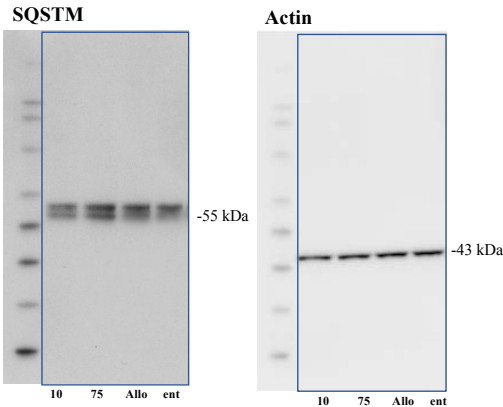

Supplement: Supplementary file 4 [file DataSheet1.PDF]
