# Supplementary material for: The Enantiomer of Allopregnanolone Prevents Pressure-Mediated Retinal Degeneration Via Autophagy
Source: Front Pharmacol. 2022 Mar 16;13:855779. doi: 10.3389/fphar.2022.855779 (PMC8966700; doi:10.3389/fphar.2022.855779)
Supplement: Supplementary file 2 [file DataSheet4.PDF]

Source data of Table 1.

Table S1. Morphological changes of retinas after pressure-loading and neurosteroids treatment.

NFLT	10 mmHg	75 mmHg	75 mmHg+ 1 μM AlloP	75 mmHg+ 1 μM *ent*- AlloP	75 mmHg+1 μM AlloP+ 1 μM Picrotoxin	75 mmHg+1 μM *ent*- AlloP+ 1 μM Picrotoxin
1	2.1	10.2	2.0	2.3	2.3	2.0
2	2	9.9	3.0	1.9	5.9	1.9
3	0.3	13.5	1.0	1.0	1.2	1.8
4	0.7	10.1	2.3	1.5	3.4	2.5
5	1	13.2	1.5	1.8	5.1	0.8
6	1.7	14	1	0.7	1.9	0.7
7	1.6	9.3	2.2	1.9	8.9	1.5
8	2.1	11	1.7	2.5	5.3	1.7
9	0.9	10.4	1	1.0	2.1	1.0
10	1	11.0	1.4	1.5	2.2	0.9
Average	1.3	11.3	1.7	1.6	3.8	1.5
SD	0.7	1.9	0.7	0.6	2.6	0.6
Dunnett's test		vs	<0.05*	<0.05*	<0.05*	<0.05*

NDS	10 mmHg	75 mmHg	75 mmHg+ 1 μM AlloP	75 mmHg+ 1 μM *ent*- AlloP	75 mmHg+1 μM AlloP+ 1 μM Picrotoxin	75 mmHg+1 μM *ent*- AlloP+ 1 μM Picrotoxin
1	1	0	0	0	4	0
2	0	0	1	1	3	0
3	0	0	0	0	2	0
4	0	1	0	1	4	1
5	0	1	0	0	4	0
6	0	1	1	0	3	1
7	1	1	0	0	3	0
8	0	1	0	0	2	1
9	0	1	0	0	3	0
10	0	1	0	1	2	0
Average	0.2	0.7	0.2	0.3	3.0	0.3
SD	0.4	0.5	0.4	0.5	0.8	0.5
Dunnett's test		vs	>0.05	>0.05	<0.05*	>0.05

Damaged cell	10 mmHg	75 mmHg	75 mmHg+ 1 μM AlloP	75 mmHg+ 1 μM *ent*- AlloP	75 mmHg+1 μM AlloP+ 1 μM Picrotoxin	75 mmHg+1 μM *ent*- AlloP+ 1 μM Picrotoxin
1	0	20	2	1	21	1
2	0	17	4	1	14	3
3	0	11	3	4	30	2
4	2	9	0	2	21	1
5	1	19	0	1	34	1
6	2	23	1	3	21	2
7	4	10	5	1	25	2
8	3	22	2	3	31	4
9	0	15	1	1	18	3
10	0	10	1	1	25	1
Average	1.3	16.2	2.0	1.9	23.9	2.1
SD	1.5	5.3	1.7	1.2	6.6	1.1
Dunnett's test		vs	<0.05*	<0.05*	<0.05*	<0.05*

Source data of Table 2.

Table S2. Morphological changes of retinas after 3-MA and neurosteroids treatment.

NFLT	75 mmHg+3-MA	75 mmHg+ 1 μM AlloP +3-MA	75 mmHg+ 1 μM *ent*- AlloP +3-MA
1	1.2	2.1	12.3
2	2.0	2.4	11.9
3	3.5	1.4	11.0
4	1.1	1.3	11.2
5	1.2	2.0	11
6	1.1	2.0	10.5
7	0.3	1.5	11.9
8	1	1.9	12.9
9	1.0	1.0	11.3
10	1.1	1.9	9.5
Average	1.4	1.8	11.4
SD	1.0	0.4	0.8
Dunnett's test	vs	>0.05	<0.05*

NDS	75 mmHg+3-MA	75 mmHg+ 1 μM AlloP +3-MA	75 mmHg+ 1 μM *ent*- AlloP +3-MA
1	2	1	1
2	1	0	2
3	2	0	1
4	1	0	1
5	1	0	1
6	2	1	2
7	1	0	2
8	1	0	1
9	2	1	2
10	1	0	1
Average	1.4	0.3	1.4
SD	0.5	0.5	0.5
Dunnett's test	vs	<0.05*	>0.05

Damaged cell	75 mmHg+3-MA	75 mmHg+ 1 μM AlloP +3-MA	75 mmHg+ 1 μM *ent*- AlloP +3-MA
1	16	2	25
2	23	5	14
3	17	2	14
4	19	7	12
5	12	6	13
6	20	7	13
7	14	5	23
8	20	3	13
9	18	7	27
10	17	3	16
Average	17.7	4.9	17.1
SD	3.4	2.1	6.0
Dunnett's test	vs	<0.05*	>0.05

Source data of figures.

Fig. 2k. RGC Survival

RGC / mm2

	10 mmHg	75 mmHg	75 mmHg+AlloP	75 mmHg+ent-AlloP
1	2987.5	573.6	2676.8	2724.6
2	2461.7	1003.8	2222.7	2390
3	2366.1	549.7	2700.7	2198.8
4	2605.1	645.3	2198.8	2222.7
5	2581.2	573.6	2652.9	2533.4
Average	2600.3	669.2	2490.4	2413.9
SD	435.0	388.6	454.2	418.5
Dunnett		vs	<0.05*	<0.05*
Tukey		vs	<0.05*	<0.05*
			vs	>0.05

Fig. 2p. TUNEL Staining

	10 mmHg	75 mmHg	75 mmHg+AlloP	75 mmHg+*ent*-AlloP
1	1	29	0	1
2	0	25	0	0
3	0	37	0	0
4	0	20	1	1
5	0	17	5	0
Average	0.2	25.6	1.2	0.4
SD	0.4	7.9	2.2	0.5
Dunnett		vs	<0.05*	<0.05*
Tukey		vs	<0.05*	<0.05*
			vs	>0.05

Fig. 3n. Autophagosomes (AP) /25 µm2 in the NFL

	10 mmHg	75 mmHg	AlloP	*ent*-AlloP
1	1	1	2	1
2	0	1	2	4
3	0	1	2	5
4	0	1	2	1
5	0	1	2	5
6	0	1	2	3
7	0	1	2	3
8	0	1	2	4
9	0	1	3	4
10	0	1	2	4
Total	1	10	21	34
Average	0.1	1.0	2.1	3.4
SD	0.3	0.0	0.0	2.0
Dunnett	vs	<0.05*	<0.05*	>0.05
	vs	<0.05*	<0.05*	>0.05
Tukey		vs	<0.05*	<0.05*
			vs	<0.05*

Fig. 3o. Degenerative autophagic vacuoles (DAV)/25 µm2 in the NFL

	10 mmHg	75 mmHg	AlloP	*ent*-AlloP
1	0	0	2	3
2	0	1	2	1
3	0	1	2	4
4	0	1	2	3
5	0	1	2	3
6	1	1	1	3
7	0	1	2	3
8	0	0	2	3
9	0	1	2	2
10	0	1	2	4
Total	1	8	19	29
Average	0.1	0.8	1.9	2.9
SD	0.3	0.4	0.0	1.1
Dunnett	vs	*p<0.05	*p<0.05	<0.05*
	vs	*p<0.05	*p<0.05	<0.05*
Tukey		vs	*p<0.05	*p<0.05
			vs	<0.05*

Fig.3p-2. LC3-II expression

LC3-II

	10 mmHg	75 mmHg	AlloP	*ent*-AlloP
1	1.00	1.94	3.17	4.91
2	1.00	2.00	2.81	5.92
3	1.00	2.41	3.63	6.00
4	1.00	1.94	3.17	4.91
Average	1.00	2.07	3.20	5.44
SD	0.02	0.25	0.36	0.63
Dunnett	vs	<0.05*	<0.05*	<0.05*
Tukey	vs	<0.05*	<0.05*	<0.05*
		vs	<0.05*	<0.05*
			vs	<0.05*

Fig.3q-2. p62 expression

p62

	10 mmHg	75 mmHg	AlloP	*ent-* AlloP
1	1.00	4.07	2.58	0.31
2	1.00	2.00	1.00	0.30
3	1.00	4.18	1.91	1.06
4	1.00	2.76	0.76	0.38
Average	1.00	3.25	1.56	0.51
SD	0.02	1.07	0.86	0.39
Dunnett	vs	<0.05*	>0.05	>0.05
Tukey	vs	<0.05*	>0.05	>0.05
		vs	<0.05*	<0.05*
			vs	<0.05*

Fig.4e-2. LC3-II expression
LC3-II

	3MA	ent
1	1.00	11.20
2	1.00	8.89
3	1.00	7.78
4	1.00	8.11
Average	1.00	9.00
SD	0.02	1.56
	vs	<0.05*

Fig.4f-2. p62 expression
p62

	3MA	ent
1	1.00	4.07
2	1.00	1.90
3	1.00	4.18
4	1.00	2.76
Average	1.00	3.23
SD	0.02	1.11
	vs	<0.05*

Fig. 5a. IOP profile
AlloP

	Pre-ope	3 days	7 days	14 days	21 days
1	9	32	25	30	29
2	9	30	30	31	28
3	10	30	42	27	26
4	10	35	30	28	30
5	10	29	29	25	28
6	9	27	33	30	27
7	11	20	32	31	30
8	11	25	28	27	21
9	11	30	26	31	25
10	10	27	30	28	30
11	9	28	26	22	20
12	10	27	28	30	28
13	9	30	20	25	28
14	9	29	20	31	29
Average	9.8	28.5	28.5	28.3	27.1
SD	0.8	3.5	5.5	2.8	3.1
Dunnett	vs	p<0.05*	p<0.05*	p<0.05*	p<0.05*

***ent*- AlloP**

	Pre-ope	3 days	7 days	14 days	21 days
1	9	22	25	31	22
2	8	32	21	20	27
3	10	27	38	35	36
4	12	20	39	41	34
5	13	32	27	25	30
6	10	25	21	22	21
7	9	33	26	21	24
8	8	36	23	20	33
9	10	28	21	35	31
10	10	20	31	29	20
11	10	25	34	20	33
12	9	15	42	31	30
13	11	30	32	33	35
14	12	23	37	32	29
Average	10.1	26.3	29.8	28.2	28.9
SD	1.5	6.0	7.3	6.9	5.3
Dunnett	vs	p<0.05*	p<0.05*	p<0.05*	p<0.05*

OHT (vehicle control)

	Pre-ope	3 days	7 days	14 days	21 days
1	8	22	30	28	26
2	9	27	32	27	22
3	9	31	25	26	27
4	10	28	20	23	22
5	11	25	17	28	28
6	12	23	28	31	30
7	12	16	25	27	28
8	10	32	27	25	30
9	9	24	28	25	26
10	9	22	30	27	22
11	9	27	26	25	30
12	11	40	33	26	25
13	11	28	34	30	31
14	10	23	29	27	29
Average	10.1	24.6	25.3	26.8	26.1
SD	1.6	4.9	5.3	2.1	3.1
Dunnett	vs	p<0.05*	p<0.05*	p<0.05*	p<0.05*

Intact control

	Pre-ope	3 days	7 days	14 days	21 days
1	10	9	10	11	11
2	9	10	10	10	12
3	10	13	10	9	10
4	10	9	11	10	9
5	10	10	11	9	10
6	11	12	12	11	11
7	10	10	9	8	10
8	11	10	11	9	10
9	10	11	10	11	10
10	12	10	11	10	10
11	10	12	9	9	10
12	13	9	12	10	9
13	10	10	10	10	11
14	12	11	10	11	10
Average	10.0	10.4	10.4	9.9	10.4
SD	0.6	1.5	1.0	0.9	1.0
Dunnett	vs	p>0.05	p>0.05	p>0.05	p>0.05

Fig. 5f. TUNEL staining

	Control	OHT	AlloP	*ent*-AlloP
1	0	8	1	0
2	1	8	0	0
3	0	12	1	0
4	0	16	0	0
5	0	11	0	1
Average	0.2	11	0.4	0.2
SD	0.4	3.3	0.5	0.4
Dunnett		vs	<0.05*	<0.05*
Tukey		vs	<0.05*	<0.05*
			vs	>0.05

Fig.5k. RGC Survival RGC / mm2

	Control	OHT	AlloP	*ent*- AlloP
1	2891.9	1218.9	2629	2461.7
2	3393.8	1481.8	2676.8	2700.7
3	2294.4	932.1	2318.3	2987.5
4	2461.7	1003.8	2222.7	2629
5	2413.9	1075.5	2151	2939.7
Average	2691.1	1142.4	2399.6	2743.7
SD	832.1	596.4	618.5	598.4
Dunnett		vs	<0.05*	<0.05*
Tukey		vs	<0.05*	<0.05*
			vs	>0.05

Fig. 5p. Density of axons in ONs (Axons/ 100 µm2)

	Control	OHT	AlloP	*ent*- AlloP
1	25	15	25	33
2	30	16	30	32
3	29	14	27	29
4	28	20	30	27
5	29	18	31	31
Average	28.2	16.6	28.6	30.4
SD	1.9	2.4	2.5	2.4
Dunnett		vs	<0.05*	<0.05*
Tukey		vs	<0.05*	<0.05*
			vs	>0.05

Fig. 6e-1
Autophagosomes (AP) /25 µm2 in the NFL

	Control	OHT	AlloP	*ent*-AlloP
1	1	1	1	2
2	0	1	1	2
3	0	1	2	2
4	0	0	1	3
5	0	1	2	1
6	0	1	2	2
7	0	1	1	2
8	0	0	1	2
9	0	0	2	3
10	0	1	1	2
Total	1	7	14	21
Average	0.1	0.7	1.4	2.1
SD	0.3	0.4	0.5	0.7
Dunnett	vs	*p<0.05	*p<0.05	*p<0.05
Tukey	vs	*p<0.05	*p<0.05	*p<0.05
		vs	*p<0.05	*p<0.05
			vs	*p<0.05

Fig. 6e-2
Degenerative autophagic vacuoles (DAV) /25 µm2 in the NFL

	Control	OHT	AlloP	*ent*-AlloP
1	0	1	2	4
2	0	1	2	4
3	0	0	1	3
4	0	1	2	3
5	1	1	2	2
6	0	1	2	3
7	0	1	1	2
8	0	1	2	3
9	0	1	2	2
10	0	0	1	3
Total	1	8	17	29
Average	0.1	0.8	1.7	2.9
SD	0.3	0.4	0.4	0.8
Dunnett	vs	*p<0.05	*p<0.05	*p<0.05
Tukey	vs	*p<0.05	*p<0.05	*p<0.05
		vs	*p<0.05	*p<0.05
			vs	*p<0.05

Fig. 6j-1
Autophagosomes (AP) /100 µm2 in the ON

	Control	OHT	AlloP	*ent*-AlloP
1	1	1	2	3
2	0	1	2	1
3	0	0	1	3
4	0	1	1	3
5	0	1	2	3
6	0	1	1	3
7	0	1	2	3
8	0	1	1	2
9	0	0	2	3
10	0	1	1	2
Total	1	8	15	26
Average	0.1	0.8	1.5	2.6
SD	0.3	0.4	0.5	0.9
Dunnett	vs	*p<0.05	*p<0.05	*p<0.05
Tukey	vs	*p<0.05	*p<0.05	*p<0.05
		vs	*p<0.05	*p<0.05
			vs	*p<0.05

Fig. 6j-2
Degenerative autophagic vacuoles (DAV) /100 µm2 in the ON

	Control	OHT	AlloP	*ent*-AlloP
1	1	0	2	2
2	0	0	1	2
3	0	1	2	2
4	0	0	2	2
5	0	1	1	3
6	0	1	2	2
7	0	1	2	3
8	0	1	1	3
9	0	1	2	3
10	0	0	1	3
Total	1	6	16	25
Average	0.1	0.6	1.6	2.5
SD	0.3	0.5	0.5	0.4
Dunnett	vs	*p<0.05	*p<0.05	*p<0.05
Tukey	vs	*p<0.05	*p<0.05	*p<0.05
		vs	*p<0.05	*p<0.05
			vs	*p<0.05

LC3-II expression (Fig.3p-2)
LC3-II

	10 mmHg	75 mmHg	AlloP	*ent*-AlloP
1	1.00	1.94	3.17	4.91
2	1.00	1.53	2.82	4.27
3	1.00	1.77	3.11	5.50
4	1.00	1.77	3.11	5.04
Average	1.00	1.75	3.05	4.93
SD	0.02	0.19	0.18	0.53
Dunnett	vs	<0.05*	<0.05*	<0.05*
Tukey	vs	<0.05*	<0.05*	<0.05*
		vs	<0.05*	<0.05*
			vs	<0.05*

p62 expression (Fig.3q-2)
p62

	10 mmHg	75 mmHg	AlloP	*ent-* AlloP
1	1.00	3.33	2.56	1.90
2	1.00	3.43	2.56	1.27
3	1.00	3.63	2.70	0.83
4	1.00	3.02	2.46	1.12
Average	1.00	3.35	2.57	1.28
SD	0.02	0.27	0.12	0.47
Dunnett	vs	<0.05*	>0.05	>0.05
Tukey	vs	<0.05*	<0.05*	>0.05
		vs	<0.05*	<0.05*
			vs	<0.05*

Fig. 7b. Positive STR

	Control	OHT	AlloP	ent-AlloP
	26.66	11.45	21.32	25.28
	28.02	14.31	32.62	22.78
	26.9	12.2	25.9	26
	27.25	11.1	26.01	30.41
Average	27.2	12.3	26.5	26.1
S.D.	0.6	1.4	4.7	3.2
